# The Genetic Perspective of Familial Glucocorticoid Deficiency: *In Silico* Analysis of Two Novel Variants

**DOI:** 10.1155/2020/2190508

**Published:** 2020-09-01

**Authors:** Katayoun Heshmatzad, Nejat Mahdieh, Ali Rabbani, Abdolah Didban, Bahareh Rabbani

**Affiliations:** ^1^Growth and Development Research Center, Tehran University of Medical Sciences, Tehran, Iran; ^2^Rajaie Cardiovascular Medical and Research Center, Iran University of Medical Sciences, Tehran, Iran; ^3^Department of Pediatrics, Pediatric Endocrinologist, Qazvin University of Medical Sciences, Qazvin, Iran

## Abstract

Familial glucocorticoid deficiency is a rare autosomal recessive genetic disorder which belongs to a group of primary adrenal insufficiency (PAI) and is mainly caused by mutations in the *MC2R* and *MRAP* genes. A comprehensive search was conducted to find the reported variants of *MC2R* and *MRAP* genes. *In silico* pathogenic analysis was performed for the reported variants. PCR amplification and sequencing were performed for three patients. Structural analysis, modeling, and interactome analysis were applied to characterize novel *MC2R* variants and their proteins. About 80% of *MC2R*-related cases showed the clinical symptoms which were diagnosed at <2 years old. 107 patients had *MC2R* mutations (85 homozygotes, 21 compound heterozygotes, and 1 simple heterozygote). 59 variants were found in the *MC2R* gene. Four mutations were responsible for half of patients. 39 homozygous patients had *MRAP* mutations; 14 variants were determined in the *MRAP* gene. Nine proteins were predicted by STRING to associate with the studied proteins. Two novel *MC2R* variants, c.128T > G (p.Leu43Arg) and c.251T > A (p.Ile84Asn), were found in two patients at the age of above and below 2 years, respectively. Mutations in *MC2R* and *MRAP* genes are the main cause of FGD. Genetic studies and *in silico* analysis will help to confirm the diagnosis.

## 1. Introduction

Familial glucocorticoid deficiency (FGD) (OMIM #202200), a type of primary adrenal insufficiency (PAI), is an autosomal recessive disorder, known as isolated glucocorticoid deficiency or hereditary unresponsiveness to adrenocorticotropic hormone (ACTH) occur during early childhood and neonatal periods [1, 2]. The prevalence of the disease is unknown, and it seems to be very rare.

ACTH regulates the adrenal cortex. Adrenocorticotropic hormone receptor (MC2R) is mostly in the zona fasciculata which controls the glucocorticoid and cortisol synthesis [3]. A defect in MC2R downregulates the cortisol synthesis and abrogates ACTH stimulation; increased level of ACTH causes skin pigmentation [2]. Clinical manifestations of FGD include a variety of signs such as hypoglycemia, jaundice, failure to thrive, hyperpigmentation of the skin, eczema, and increased susceptibility to infection [4, 5]. If not treated, it leads to death. The FGD diagnosis is based on biochemical tests including low cortisol level, high ACTH level, normal aldosterone, and normal renin level [2].

FGD is a genetically heterogeneous disorder consisting of more than two subtypes including FGD type 1 (OMIM 607397) caused by mutations in the *MC2R* gene which constitutes about 25% of FGD cases, and FGD type 2 (OMIM 609196) consisting the mutations in *MC2R* accessory protein (*MRAP* gene), accounting for 20% of patients [6]. Recently, next-generation sequencing has revealed the causes of other types of FGD [7–13]. Genetic testing is used to confirm clinical diagnosis and distinguish various subtypes of PAI.

Different types of *MC2R* and *MRAP* mutations including missense/nonsense, regulatory, small insertions, and deletion mutations have been reported to cause FGD in the human genetic mutation database (HGMD professional 2019, http://www.hgmd.cf.ac.uk).

Here, the spectrum of the *MC2R* and *MRAP* variants, positional effect of the variants, and pathogenic analysis are determined for all the reported variants. Genotype-phenotype correlation was investigated for these variants. In addition, novel variants in *MC2R* are also reported in this study. Structural analysis and modeling were performed for the reported novel variants to determine their pathogenicity.

## 2. Materials and Methods

### 2.1. Case Presentation

**Case 1 .**

A 5-month-old infant girl who was born with cesarean section due to breach presentation with normal Apgar score was the first child of a healthy family with consanguineous marriage. Other family members did not have history of any disease. Her height, weight, and head circumference were 50 cm, 3300 g, and 37 cm at birth, respectively; all were within the normal range.

At 17^th^ day of birth, she was admitted to the newborn intensive care unit (NICU) due to hypoglycemia and seizure. Her blood glucose was 2 mmol/L (normal range: 2.5–5.5 mmol/L). Biochemical investigations showed high thyroid-stimulating hormone (TSH) level of 14.2 U/L (normal range: 0.5–5 U/L), growth hormone (GH) level of 13 ng/ml (normal range: > 10 ng/ml), insulin level of 1.43 mU/L (normal range: < 2 mU/L), and cortisol level of 495 nmol/L (normal range: > 497 nmol/L). Autoimmune analysis was not performed for the patient since she was a newborn. Physical examination showed mild skin pigmentation and normal female external genitalia. She was discharged from the hospital with levothyroxine (10 mg/kg/day), and blood glucose was stabilized.

By 4^th^ month of age checkup, weight was 5.3 kg (25^th^ percentile), height was 61.5 cm (50^th^ percentile), and head circumference was 41.5 cm (50^th^ percentile). There was no documentation on recurrent infection. Biochemical investigations showed a very low cortisol level of <0.054 *μ*g/dL (normal range: 7–25 *μ*g/dL), very high ACTH level of >2000 pg/mL (normal range: <50 pg/mL), normal aldosterone level of 866 pg/mL (normal range: 20–1100 pg/mL), normal 17-hydroxyprogesterone (17-OHP) level of 0.74 ng/mL (normal range: <1 ng/mL), and TSH level of 0.5 U/L (normal range: 0.5–5 U/L). Blood electrolytes were at normal range. Upon physical examination, hyperpigmentation was obvious with female external genitalia, and neurological development was normal. She was suspicious of a type of PAI. We started oral hydrocortisone (15 mg/m^2^/day).

At six‐month‐old, biochemical assays showed 17OHP < 0.1 ng/mL (normal range: <1 ng/mL) and TSH 5.3 U/L. She was 5.9 kg (3rd percentile), with height 64.3 cm (15^th^ percentile). She underwent genetic testing to confirm FGD diagnosis.

At the age of 15 months, (9^th^ month of treatment), her weight was 14 kg (97^th^ percentile), height was 80 cm (80^th^ percentile), and head circumference was 47 cm (85^th^ percentile); TSH level was 0.61 U/L (normal range: 0.5–5 U/L), and ACTH level was 1310 pg/ml (normal range: <50 pg/mL). She was in good condition.

**Case 2 .**

A 2-year-old boy who was born from a first-cousin couple referred for genetic testing of congenital adrenal hypoplasia and FGD. He had hypoglycemia when he was 14 months old. Other family members did not have history of any disease. His brother, aunts, and uncles did not show the disease; they were analyzed for genetic variants.

His blood glucose was 2.2 (normal range: 2.5–5.5 mmol/L). Biochemical investigations showed a high TSH level of 12 U/L (normal range: 0.5–5 U/L), GH level of 4 ng/ml (normal range: > 10 ng/ml), insulin level of 1.5 mU/L (normal range: < 2 mU/L), ACTH level of 118 pg/mL (normal range: <50 pg/mL), aldosterone level of 900 pg/mL (normal range: 20–1100 pg/mL), 17-OHP level of 1.3 ng/mL (normal range: <1 ng/mL), and cortisol level of 250 nmol/L (normal range: >497 nmol/L). Autoimmune analysis was not performed for the patient since he was young. Physical examinations showed mild skin pigmentation. He had hypothyroidism and congenital adrenal hypoplasia. He underwent hydrocortisone treatment. He had responded well to the conventional treatment.

**Case 3 .**

A 15-year-old girl born from a first-cousin couple was referred to confirm genetic diagnosis of FGD. Her parents mentioned the history of hypoglycemia at the 3^rd^ day of birth and hospitalization. Other family members did not have history of any disease. Her brother was also healthy and had no sign. At the 2^nd^ year of life, physical examination showed skin pigmentation and normal female external genitalia.

Her blood glucose was 2.5 (normal range: 2.5–5.5 mmol/L). Biochemical investigations showed a GH level of 6 ng/ml (normal range: > 10 ng/ml), insulin level of 1.5 mU/L (normal range: < 2 mU/L), ACTH level of 282 pg/mL (normal range: <50 pg/mL), aldosterone level of 700 pg/mL (normal range: 20–1100 pg/mL), 17-OHP level of 3 ng/mL (normal range: < 1 ng/mL), and cortisol level of 200 nmol/L (normal range: >497 nmol/L). No autoimmune analysis was performed for the patient. She was clinically diagnosed as FGD. She underwent hydrocortisone treatment and did well until the genetic testing. She underwent molecular testing to confirm the diagnosis at later ages.

### 2.2. Molecular Genetic Analyses

Informed consent was obtained from patients and the parents or guardian of the minors for genetic testing. DNA was extracted according to the standard methods. In brief, PCR amplification was performed using routine protocols; direct sequencing was performed by the sequencing analyzer (ABI3130XL, Biosystems, US) for coding regions of *MC2R* and *MRAP* genes.

### 2.3. Data Abstraction

A comprehensive search was conducted on published variants in PubMed using keywords including “MC2R gene mutations [ti/abs]” and “Familial glucocorticoid deficiency AND MC2R.” MRAP variants were identified based on keywords including “MRAP gene mutations [ti/abs]” and “Familial glucocorticoid deficiency AND MRAP.”

Articles including functional studies, animal studies, and mechanism of disease were excluded from our study. According to our inclusion criteria, articles having FGD patients with defined *MC2R* and/or *MRAP* variants were extracted. The variants were compared to HGMD Professional 2020 to ensure all the selected variants. Clinical phenotype, total recruited patients, number of FGD cases, protein change, nucleotide change, and zygosity were extracted from the articles.

### 2.4. Characterization of the *MC2R* and *MRAP* Variants

All variants were named based on the human genome variation (HGVS) nomenclature, and the type of mutation, positional effect, and functional effect were used for further study. *In silico* analysis was performed for the variants. The position of amino acids, domain, and motif was based on UniProtKB/Swiss-Prot Q01718.

## 3. *In Silico* Analysis

### 3.1. SNP Annotation

The pathogenic significance of selected mutations was analyzed based on reference sequence NM_000529 and NP_000520.1 (*MC2R* gene) and NM_206898.1 and NP_996781.1 (*MRAP* gene) and by different software tools including MutationTaster (http://www.mutationtaster.org/), PolyPhen-2 (Polymorphism Phenotyping) (http://genetics.bwh.harvard.edu/pph2/), PROVEAN (Protein Variation Effect Analyzer) (http://provean.jcvi.org/index.php), and CADD (Combined Annotation-Dependent Depletion) [14–17].

### 3.2. Structural Analysis and Modeling

A multiple sequence alignment was carried out in UniProtKB for the MC2R protein to demonstrate the conservation in different paralogs and orthologs within the position of the studied cases.

Human MC2R protein, known as adrenocorticotropic hormone receptor, is a member of G-protein melanocortin receptor. There is no experimentally structured model for MC2R. We conducted protein modeling based on SWISS-model (https://www.swissmodel.expasy.org). The structural analysis was based on the structured models.

The structural analysis was performed based on Phyre2 and DynaMut [18,19].

### 3.3. Interactome Analysis

Interaction of the encoded protein of MC2R and MRAP with other proteins was predicted by STRING-10, a protein-protein interaction system analysis (V10.5) [20].

### 3.4. Statistical Analysis

Data were analyzed by Statistical Package for Social Sciences (SPSS version 22.0, SPSS, Inc., Chicago, Ill, USA).

## 4. Results

### 4.1. Molecular Analyses

**Figure 1:**
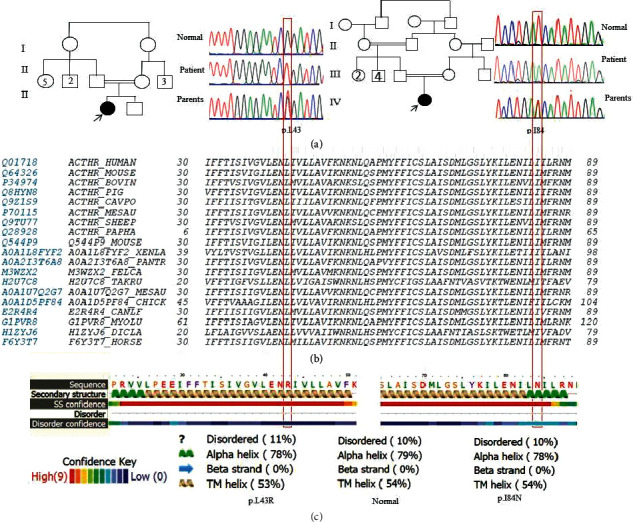
(a) Electropherogram of the patients having novel variants shows homozygote variants at positions c.128T > G and c.251T > A in the *MC2R* gene, and segregation analysis of the parents is shown. (b) Alignment of the *MC2R* gene at positions 43 and 84 in different species shows conservation. (c) Phyre structural analysis for p.Leu43Arg and p.Ile84Asn based on disordered and secondary structure effects. The helix does not change, and the influence is very low. The order is changed for p.Leu43Arg more than p.Ile84Asn.

Three homozygous mutations including two novel mutations (c.128T > G and c.251T > A) were found in the studied patients (Figure 1(a), Supplementary Materials, Table S1). The other variant was c.697G > C (p.Ala233Pro). Pedigree and segregation analysis showed that healthy family members were heterozygote for the variant in each affected family (Figure 1(a)).

*In silico* pathogenicity analysis of the variants c.128T > G (p.Leu43Arg) and c.251T > A (p.Ile84Asn) predicated to be disease-causing variants by MutationTaster, probably damaging by PolyPhen-2, deleterious by PROVEAN, and deleterious and pathogenic by CADD (PHRED score 25 for both variants) (Supplementary Materials, Table S1).

### 4.2. Alignment Analysis Modeling and Structural Analysis

Alignment of the MC2R protein at positions 43 and 84 showed conservation in different species (Figure 1(b)).

**Figure 2:**
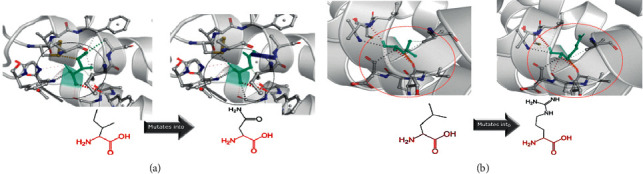
(a) Substitution of p.Ile84Asn in the *MC2R* gene: the left panel is the wild type, and the right panel is mutant asparagine. The interaction of the amino acids was changed in the substitutions. (b) Substitution of p.Leu43Arg in the *MC2R* gene and amino acid interaction change. Characterization of the reported *MC2R* and *MRAP* variants.

The modeling was based on structural experiment 6jzh (adenosine receptor A2a, soluble cytochrome b562) with coverage of amino acids 20 to 295 and identity of 27.82% for position Ile84. No structural damage was detected based on isoleucine (hydrophobic) to asparagine (hydrophilic) substitution which does not trigger any change; both amino acids are exposed to uncharged amino acid replacement. Only the interactions are influenced by the substitution (Figure 2(a)).

The modeling for Leu43 was based on 5tgz (cannabinoid receptor 1, flavodoxin) with the identity of 29.07% and coverage of amino acids of 26 to 291.

Based on the free Gibbs energy (ΔΔG) prediction, it was 1.42 kcal/mol and showed a stabilizing structure for Ile84Asn but decrease of molecule flexibility. The structural change based on ΔΔG for Leu43Arg was 0.711 kcal/mol which showed a stabilizing structure. It also decreased the molecule flexibility. Also, structure interaction changes were influenced by amino acid change (Figure 2(b)). Phyre structural analysis was predicted to be disordered with low confidence score (Figure 1(c)). Overall, Phyre predicted no structural damage for two variants.

### 4.3. Characterization of the Reported MC2R and MRAP Variants

Our search strategy yielded 84 articles including original, case reports, and reviews. After exclusion, 33 articles were related to the *MC2R* gene. 495 patients were studied which had different PAI disorders including FGD, unresponsiveness ACTH, Allgrove syndrome, and triple A syndrome. 107 cases were genetically diagnosed as FGD.

**Figure 3:**
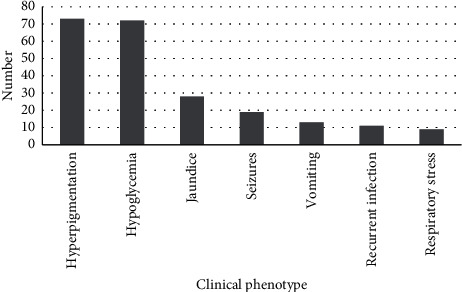
Frequency of the phenotypic characterization of the reported variants in the literature.

The clinical phenotype and presentation were available for 93 of the patients. About 80% of MC2R-related cases were presented at the age of <2 years (data not shown). All these patients had high levels of ACTH, and 79% of patients (73) had hyperpigmentation as the major sign of FGD; 77% showed hypoglycemia (72), 20% had seizures (19), 30% had jaundice (28), 14% had vomiting (13), 10% had respiratory stress (9), and 12% had recurrent infection (11) (Figure 3).

107 of FGD patients had *MC2R* mutations; 85 homozygotes, 21 compound heterozygotes, and one heterozygote of the *MC2R* gene were defined. The frequency of homozygous genotypes was 79% (85 of 107) (Supplementary Materials, Table S1).

Totally, 59 variants were found in the *MC2R* gene. Among 59 mutations, 42 were missenses, 3 were nonsenses, 13 were frameshifts, and one was regulatory variant (Supplementary Materials, Table S2). Two articles did not have enough information about the zygosity and number of patients [21, 22]. Four mutations, c.221G > T, c.560delT, c.450_460insC, and c.437G > C accounted for 49% of *MC2R*-related patients; their frequencies were 27% (56 of 207), 10%, 6%, and 6%, respectively. c.221G > T (p.Ser74Ile) was homozygous in 25 patients and compound heterozygous in 6 cases. c.560delT (p.Val187Alafs*∗*29) was reported in 10 homozygous patients and 1 compound heterozygous patient (Supplementary Materials, Tables S1 and S2). In addition, nine *MRAP* articles were included in our study. Totally, 39 *MRAP* homozygous mutations were reported in 40 patients (Supplementary Materials, Table S1). Patients with the *MRAP* variant included 3 deletions, 6 splice sites and intronics, 4 missenses, and one nonsense (Supplementary Materials, Table S2). Two mutations, c.106 + 1delG (24 of 78, 31%) and c.3G > A (Met1Ile) (18 of 78, 23%), had more frequency compared to the remaining variants. These two mutations together constituted more than half of the *MRAP* mutations (53%). Pathogenicity of the variants was investigated to ensure the causative effect (Supplementary Materials, Table S2); however, the functional effect of the missense variants has been investigated for some of the variants (Supplementary Materials, Table S2).

**Figure 4:**
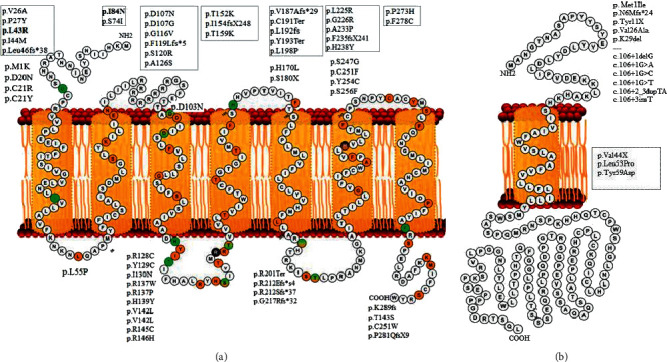
(a) MC2R receptor has 297 amino acids and consists of 7 transmembrane domains from positions 24–49 (TM helix 1, 26–52), 59–79 (TM helix 2, 59–85), 105–126 (TM helix 3, 103–133), 148–168 (TM helix 4, 145–165), 181–199 (TM helix 5, 170–199), 218–244 (TM helix 6, 212–242), and 257–278 (TM helix 7, 255–280). This receptor consists of four cytoplasmic domains expanded in positions 50–58, 127–147, 200–217, and 279–297 and four extracellular domains 1–23, 80–104, 169–180, and 245–256. (b) MRAP consists of 172 amino acids, and positions 38–58 are located in the transmembrane domain. Reported variants of each domain and region are shown.

*MC2R* variants expanded throughout the protein in seven transmembrane helixes (*n* = 27), three extracellular regions (*n* = 12), and three cytoplasmic (*n* = 18) regions. The distribution of the variants was determined on the scheme (Figure 4(a)). The position of the *MRAP* variants also showed 43% (Figure 4(b)).

### 4.4. Interactome Analysis

**Figure 5:**
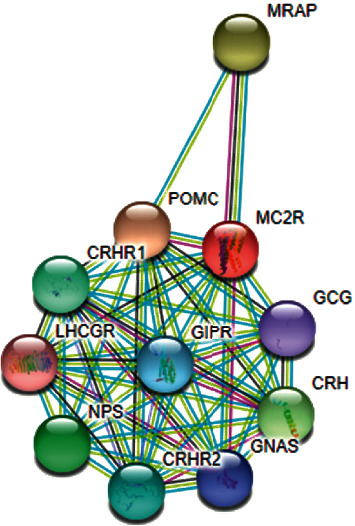
Interactome analysis by STRING (V10.5). Protein-protein interaction network; predicted functional partners are as follows: POMC: proopiomelanocortin, MC2R: melanocortin-2 receptor, CRH: corticotropin-releasing hormone, GNAS: GNAS complex locus, CRHR2: corticotropin-releasing hormone receptor 2, NPS: neuropeptide S, LHCGR: luteinizing hormone/choriogonadotropin receptor, CRHR1: corticotropin-releasing hormone receptor 1, and MRAP: melanocortin-2 receptor accessory protein.

MC2R and MRAP proteins predicted to associate with nine proteins as follows: POMC: proopiomelanocortin, MC2R: melanocortin-2 receptor, CRH: corticotropin-releasing hormone, GNAS: GNAS complex locus, CRHR2: corticotropin-releasing hormone receptor 2, NPS: neuropeptide S, LHCGR: luteinizing hormone/choriogonadotropin receptor, CRHR1: corticotropin-releasing hormone receptor 1, and MRAP: melanocortin-2 receptor accessory protein (Figure 5). Interactome analysis showed the association of the *MC2R* gene with *MRAP*.

The genes interacting with the adrenocorticoid receptor are shown in Figure 5. Therefore, overlapping phenotypes may be seen because many genes are involved. The enzymes and proteins mentioned may act in different pathways, therefore showing variable phenotypes.

## 5. Discussion

The genetic study of FGD is of importance. FGD is a clinical heterogeneous disorder which could lead to death if remains untreated; for example, recurrent hypoglycemia leads to mental disability which is categorized as lethal [23].

Mutations in *MC2R* and *MRAP* genes account for approximately 45% of FGD causes [6]. Single-gene analysis, as a cost-benefit test, could be used for confirming clinical diagnosis and distinguishing various subtypes of PAI, although next-generation sequencing has revealed the causes of other types of FGD [7–13].

In brief, our systematic analysis showed that *MC2R* and *MRAP* mutations are responsible for 22% and 8% of PAI patients (107 and 40 of 495 cases), respectively. Overall, 30% of the variants were detected by single-gene analysis (Supplementary Materials, Table S2). According to HGMD, 56 mutations have been reported for *MC2R*, but we have found 59 variants; in addition, two novel mutations were reported in this study.

Interestingly, our literature review revealed no splice or intronic variant, found to cause FGD1, while more than half of FGD2 patients (44 of 78) were due to these types of mutations. c.221G > T (p.Ser74Ile) and c.560delT (p.Val187Alafs*∗*) have high frequencies in some cohorts due to mutational hotspots; however, further studies are required using haplotype analysis to rule out the role of founder effect phenomenon. c.221G > T was found in 29% of patients (31 of 107). In a study, Chan et al. reported 34 families with *MC2R* mutations in which 16 of them had c.221G > T. They did not mention the ethnicities or geographical locations of these families [24]. In the same way, Guran et al. reported c.560delT in 9 of 23 studied families with *MC2R* mutations, but they did not provide information of patients' ethnicities [25]. c.106 + 1G in *MRAP* is a mutational host spot; it is found with different nucleotide substitutions: c.106 + 1G > A, c.106 + 1G > C, c.106 + 1G > T, and c.106 + 1delG (Supplementary Materials, Table S1).

In addition, 2 novel mutations in 2 Iranian families are reported for the first time in patients with different age of onset. Our studied patients had hypothyroidism and hyperpigmentation. Based on prediction analysis, these variants do not make significant structural changes in the protein. The mutations p.Leu43Arg and p.Ile84Asn are located in TM1 and TM2, respectively. They may result in loss of signal transduction rather than structural disruption or ligand binding on the transmembrane protein. Regarding *in silico* structural analysis, these substitutions do not impair the structure.

Our results determined that the rate of homozygosity is high (85 of 107). Also, the study indicated that 46% *MC2R* mutations were in the transmembrane domain. Despite the distribution of *MC2R* mutations, most of *MRAP* mutations were intronic mutations (6 of 14) which affect the extracellular domain (Figure 4).

In 2014, Meimaridou et al. conducted a review article on genetics of FGD and collected mutations in different genes involved in FGD including *MC2R*, *MRAP*, *NNT*, *STAR*, *CYP11A*, *MCM4*, and *AAAS* [26]. In another study by Lim, three affected FGD patients were reported, and no genetic analysis was conducted, compared with our study [27]. This literature analysis has determined more variants in comparison to the study performed by Abuduxikuer et al. in 2019 [28].

The enzymes and proteins shown in the interactome analysis may be a plausible explanation that patients show variable phenotypes. This makes genotype-phenotype correlation difficult to explain. As observed in the studied cases, different presentations might be due to different interactions, modifier genes, and variable expressions at different stages of life compared to other reported patients. Also, type of the variant may complicate the phenotype. Various overlapping phenotypes could be expected due to interactions of different proteins.

Our study was limited to a small number of studies in the genetics of FGD. FGD is a rare disorder according to Orphanet (https://www.orpha.net/consor/cgi-bin/index.php). We cannot estimate the exact frequency of FGD because this is a rare disorder, and only a few number of isolated cases were reported in case report studies. The clinical information was not available for all studies. Some articles did not have enough data including biochemical assays. Other genes such as *NNT* and *STAR* involved in FGD were not reported in this study, and only the two major genes *MC2R* and *MRAP* were investigated in this study. These two genes were not studied for all the cases, and depending on clinical evaluation, genes were selected for genetic testing; this may be the reason for differences in the diagnosis rate of FGD.

## 6. Conclusion

In conclusion, four *MC2R* and two *MRAP* mutations are responsible for half of FGD1. These mutational hot spots should be considered in screening programs in each region. Genetic testing is needed to confirm the clinical diagnosis of FGD. Treatment of glucocorticoids is adjusted by drug dosage which prevents hypoglycemia and allows normal growth and development.

## Data Availability

No other data were used to support this study.

## Supplementary Materials

Supplementary Materials Supplementary Materials: information on genetic characterization of the reported *MC2R* and *MRAP* gene variants.
