# Supplementary material for: The Genetic Perspective of Familial Glucocorticoid Deficiency: In Silico Analysis of Two Novel Variants
Source: Int J Endocrinol. 2020 Sep 1;2020:2190508. doi: 10.1155/2020/2190508 (PMC7481914; doi:10.1155/2020/2190508)
Supplement: Supplementary Materials — information on genetic characterization of the reported MC2R and MRAP gene variants. [file 2190508.f1.docx]

**The genetic perspective of familial glucocorticoid deficiency: in silico analysis of two novel variants**

Katayoun Heshmatzad*^a^, Nejat Mahdieh*^a,b^, Ali Rabbani^a^, Abdolah Didban^c^, Bahareh Rabbani^a^

a. Growth and development research center, Tehran University of Medical Sciences, Tehran, Iran

b. Rajaie Cardiovascular Medical and Research Center, Iran University of Medical Sciences, Tehran, Iran

c. Department of Pediatrics, Pediatric Endocrinologist, Qazvin University of Medical Sciences, Qazvin, Iran

Supplementary Table1: Included articles with their description, clinical information, variants, protein change, zygosity of MC2R gene

| Number | Clinical phenotype | Other diagnosed phenotype | recruited patients | Nucleotide change | Protein change | mutated patients | Zygosity | Reference |
| --- | --- | --- | --- | --- | --- | --- | --- | --- |
| 1 | FGD | adrenal hypo- and hyperplasia, Triple A, and autoimmune polyendocrinopathy syndrome type I | 17 | 1.c.424G>T  2.c.80C>G 3.433C>T | 1.Val142Leu 2.P27R 3.Arg145Cys | 1  1 | Homo  Hetero  Hetero | (1) |
| 2 | FGD | Febrile seizures shock, Hypoglycemia, Hyponatremia, mild Hyperpigmentation, coma,hypocortisolemia | 1 | 1.c.319G>A | 1.p.Asp107Asn 2.p.Pro281GlnfsX9 | 1 | Hetero | (2) |
| 3 | FGD1 | salt-losing forms of adrenal hypoplasia | 22 | 1.c.221G>T 2.437G>C,  3.560delT  4.579-.581delTGT | 1.Ser74Ile 2.Arg146His,  3.Val187Alafs*29 4.Tyr193Ter | 1 | 1.Homo 2.compond Hetero 3.Homo | (3) |
| 4 | FGD | poor sucking, moderate axial hypotonia, hyporeactivity and vomiting. During clinical evaluation, a generalised seizure occurred and was followed by desaturation, laboured breathing, generalised hypotonia and lethargy. | 1 | c.761A>G | Tyr254Cy | 1 | Homo | (4) |
| 5 | PAI | hyperpigmentation, salt-wasting crisis/electrolyte imbalance hypoglycemia with/without convulsions , vomiting/abdominal pain , prolonged jaundice, fatigue , neonatal respiratory distress), frequent infections and failure to thrive or weight loss,DSD | 95/77 genetic diagnosis | c.307G>A c.347G>T c.409C>T c.427G>T NA c.674T>G NA c.697G>C NA c.560delT Deletion(Whole exon) | Asp103Asn Gly116Va Arg137T V142L T143S L225R G226R Ala233Pro Cys251Trp p.V187Afs*29 | Total:25 mutations | Homo | (5) |
| 6 | Primary Cortisol Deficiency and Growth Hormone Deficiency | Hypoglycemia | 1 | c.634delA | p.R212Efs*s4 | 1 | Homo | (6) |
| 7 | FGD |  | 1 |  | p.SER74Ile | 1 | Homo | (7) |
| 8 | adrenal unresponsiveness to ACTH | drenal crisis despite poor drug compliance, poor pubic hair development (Tanner stage 2), well-developed breasts (Tanner stage 5), and regular menstrual cycles. | 1 | c.307G>A  c.409C>T | Asp103Asn  Arg137Trp | 1 | Hetero | (8) |
| 9 | FGD1 | hyperbilirubinemia and hyperpigmentation/ low cortisol and high ACTH levels with normal serum electrolytes and renin-aldosterone axis | 1 | c.674T>G | p.Leu225Arg | 1 | Homo | (9) |
| 10 | FGD1 | hypoglycemia, seizure, skin hyperpigmentation, hyperbilirubinemia, cholestasis, and a tall stature | 1 | c.433C>T  c.712C>T | p.R145  p.H238Y | 1 | Hetero | (10) |
| 11 | FGD | hyperpigmentation, hypoglycemia, failure to thrive, and recurrent infections | 1 | 635insC | I154H | 1 | Homo | (11) |
| 12 | FGD | Severe hypoglycemia, unmeasurable cortisol, and grossly elevated ACTH but normal electrolyte | 1 | 866dupA | Met290Aspfs*60 | 1 | Homo | (12) |
| 13 | FGD1 | FGD | 164 | 1.c.221G>T  2.c.459–460insC | 1.S74I 2.p.I154fsX248 | 42 of 34 families |  | (13) |
| 14 | ACTH resistance syndrome | chalasia and alacrima, besides the symptoms of adrenal insufficiency | 5 | c.347G>T | p.Gly116Val | 1 | Homo | (14) |
| 15 | FGD |  | 3 | c.427G>T  c.697G>C  c.360C>G | Val142Leu  Ala233Pro  Ser120Ar | 3 | Homo | (15) |
| 16 | Familial Glucocorticoid Deficiency | jaundice , hyperpigmentation | 1 | c.307G>A | D103N | 1 | Homo | (16) |
| 17 | familial glucocorticoid deficiency | skin hyperpigmentation muscle weakness, mild jaundice and constipation. Hormonal analyses revealed high ACTH and TSH serum concentrations, low serum cortisol concentration along with normal blood electrolytes | 1 | 137delT  c.145G>A | p.Leu46fs*38 and p.Val49Met | 1 | Hetero | (17) |
| 18 | FGD | thyroid dysfunction and growth hormone deficiency (GHD) | 5 | c.459_460insC | p.I154fsX248 | 5 | Homo | (18) |
| 19 | FGD | hypertonic seizures associated with hypoglycemia, skin hyperpigmentation, muscle weakness and mild jaundice. Hormonal analyses revealed high ACTH, low serum cortisol along with normal blood electrolytes | 1 | c.320A>G,c.433C>T | p.D107G,p.R145C | 1 | Hetero | (19) |
| 20 | FGD | isolated | 1 | 652-653insA | Gly217fs,  Promoter(-1017/44 bp) | 1 | Hetero | (20) |
| 21 | FGD | hypoglycemia after prolonged fasting during a respiratory tract infection | 1 | c.455C>A | T152K | 1 | Homo | (21) |
| 22 |  | FGD | 4 | c.221G>T  c.382C>T  c.132C>G  c.577-78delTA  c.437G>C | S74I  R128C  144M  L192fs  R146H | 4 | Homo | (22) |
| 23 | FGD | pneumonia and sepsis, collapse, bradycardia and hypotension at 8 months of age | 1 | c.833T>G  c.386A>G | F278C  Y129C | 1 | Homo | (23) |
| 24 | PAI/FGD |  | 63 | c.307G>A  c.676G>A | p.Asp103Asn  p.Gly226Arg | 1 FGD mutated | Hetero | (24) |
| 25 | FGD |  | 2 | c.476C>G | T159K  A233D  T159K | 2 |  | (25) |
| 26 | FGD | tall stature and skin pigmentation, | 1 | c.62G>A  c.437G>C | C21Y  R146H | 1 | Hetero | (26) |
|  |  |  |  |  |  |  |  |  |
| 27 | FGD |  | 1 | InsA1347  c.376G>T | G217fs  Ala126DSer | 1 | Hetero | (27) |
| 28 | HGD |  | 4 | c.221G>T  c.818C>A | Ser74Ile  Pro273His | 2 | Homo/Compound Hetero | (28) |
| 29 | FGD |  | 1 | c.761A>G | Tyr254cys | 1 | Homo | (29) |
| 30 | FGD |  | 2 | c.319G>A  c.752G>T  G217fs | Asp107ASn  Cys251Phe | 2 | Homo,CompondHetro | (30) |
| 31 |  | aphthous stomatitis and glossitis | 1 | c.61T>C  c.742A>G | C21R  S247G | 1 | Homo | (31) |
| 32 | HGD |  | 1 | c.601C>T  c.360C>G | Arg201Ter  Ser120Arg | 1 | ,CompondHetero | (32) |
| 33 | FGD | tall stature | 1 | c.437G>C | R146H | 1 | Homo | (33) |
| 34 | FGD |  | 11 |  | R146H  R146H  S74I/R128C  S74I  S74I  S74I | 1  1  1  1  1  1 | Homo  Homo  CH  Homo  Homo  Homo | (34) |
| 35 | FGD |  |  | c.476C>G  c.476C>G/ c.307G>A  c.221G>T/1052delC  c.221G>T/c.221G>T |  | 2  1  1  1 | Homo  CH  CH  Homo | (35) |
| 36 | FGD |  |  | c.221G>T/c.476C>G |  | 1 | CH | (36) |
| 37 | FGD |  |  | S74I/S74I  S74I/S74I  S74I/S74I  S74I/S74I S74I/R128C I44M/L192fs P27R/Normal |  | 1  1  1  1  1  1  1 | Homo  Homo  Homo  Homo  CH  CH  Hetero | (37) |

Continued supplementary Table 1- The genetic characteristics, number of patients, protein change, zygosity of MRAP gene variants in literature including

| Number | Clinical phenotype | Total recruited patients | Nucleotide change | Protein change | mutated MRAP patients | Zygosity | Reference |
| --- | --- | --- | --- | --- | --- | --- | --- |
| 1 | FGD | 1 | c.130delG | V44X | 1 | Homo | (38) |
| 2 | FGD | 1 | c.17_23del | L31X | 1 | Homo | (39) |
| 3 | FGD | 106 | c.106+1G>A | NA | 1 | Homo | (40) |
|  |  |  | c.106+1G>T | NA | 1 | Homo |  |
|  |  |  | c.106+1G>C | NA | 2 | Homo |  |
|  |  |  | c.106+1delG | NA | 5 | Homo |  |
|  |  |  | c.106+3insT | NA | 3 | Homo |  |
|  |  |  | c.3G>A | M1I | 8 | Homo |  |
|  |  |  | c.128delG | V44X | 1 | Homo |  |
| 4 | FGD2 | 1 | c. 106+1delG | NA | 1 | Homo | (41) |
| 5 | FGD | 1 | c.106+1delG | NA | 1 | Homo | (42) |
| 6 | FGD | 95 | c.158T>C | Leu53Pro | 1 | Homo | (43) |
|  |  |  | c.106+3insT |  | 1 | Homo | (43) |
|  |  |  | c.106+1delG |  | 5 | Homo | (43) |
|  |  |  | c.88_90delAAG | p.K30del | 2 | Homo | (43) |
| 7 | late-onset FGD | 2 | c.175T>G | pY59D | 1 | Homo | (44) |
|  |  |  | c.76T>C | p.V26A | 1 | Homo | (44) |
| 8 | FGD | 1 | c.106+2_3dupTA | NA | 1 | Homo | (45). |
| 9 | FGD | 5 | c.3G>A | M1I | 1 | Homo | (46) |
| 10 | Abstract | NA | c.33C>A | Y11X | 39 |  | (47) |

**Supplementary Table 2: Genetic characterization of the reported MC2R and MRAP gene variants**

| No. | Variant name | | Reported No. | Location | Mutation Taster | Polyphen-2 | Provean | CADD | Functional research reports | Ref |
| --- | --- | --- | --- | --- | --- | --- | --- | --- | --- | --- |
|  | cDNA | Amino acid |  |  |  |  |  |  |  |  |
|  | MC2R |  |  |  |  |  |  |  |  |  |
|  | Indels |  |  |  |  |  |  |  |  |  |
| 1 | Whole gene deletion | NA | 6 |  | NA | NA | NA | NA |  | (43) |
| 2 | c.137delT | p.Leu46fsX38 | 1 | TM1 | DC | NA | NA | 27.6 |  | (48) |
| 3 | c.357delC | p.Phe119LeufsX5 | 1 | Cytoplasmic | DC | NA | NA | 25.2 |  | (35) |
| 4 | c.459_460insC | p.I154HisfsX95 | 12 | TM4 | DC | NA | NA | 24.1 |  | (13), (18) |
| 5 | c.560delT | p.Val187AlafsX29 | 21 | TM5 | DC | NA | NA | 28.1 |  | (5), (3) |
| 6 | c.577-78delTA | p.Leu192fs | 2 | TM5 | DC | NA | NA | 34 |  | (22), (37) |
| 7 | c.579-581delTGT | p.Tyr193Ter | 2 | TM5 | DC | NA | NA | 20.3 |  | (3) |
| 8 | c.634delA | p.Arg212fsX215 | 4 | Cytoplasmic | DC | NA | NA | 18.88 |  | (6),(13) |
| 9 | c.635insC | p.Ile154His | 2 | TM6 | DC | NA | NA | 23.4 |  | (11) |
| 10 | c.652-653insA | p.Ala218Aspfs*31 | 1 | Cytoplasmic | DC | NA | NA | 35 |  | (20) |
| 11 | c.702delC | p.Phe235LeufsX7 | 2 | TM6 | DC | NA | NA | 35 |  | (49) |
| 12 | c.842delC | p.Pro281GlnfsX9 | 1 | Cytoplasmic | DC | NA | NA | 34 |  | (35) |
| 13 | c.866insA | p.Met290AspfsX60 | 3 | Cytoplasmic | DC | NA | NA | 29.7 |  | (35),(12) |
|  | Regulatory |  |  |  |  |  |  |  |  |  |
| 14 | c.-1017del44 | NA | 1 | NA | NA | NA | NA | NA |  | (20) |
|  | Missense/nonsense |  |  |  |  |  |  |  |  |  |
| 15 | c.2T>A | p.Met1Lys | NA | Extracellular | DC | PD | N -0.227 | 23.0 |  | (50) |
| 16 | c.58G>A | p.Asp20Asn | NA | Extracellular | DC | B | N -1.079 | 19.35 |  | (51) |
| 17 | c.61T>C | p.Cys21Arg | 1 | Extracellular | DC | PD | D-6.958 | 41 |  | (31) |
| 18 | c.62G>A | p.Cys21Tyr | 1 | Extracellular | DC | PD | D-6.26 | 24.4 |  | (26) |
| 19 | c.80C>G | p.Pro27Tyr | 1 | Cytoplasmic | DC | PD | D-4.661 | 25.1 |  | (1) |
| 20 | c.128T>G | p.Leu43Arg | 2 | TM1 | DC | PD | D-4.663 | 24.7 |  | This study |
| 21 | c.132C>G | p.Ile44Met | 2 | TM1 | P | B | N-0.088 | 7.76 | (52) | (22), (37) |
| 22 | c.145G>A | p.Val49Met | 1 | TM1 | DC | PD | N-2.305 | 25.3 |  | (17) |
| 23 | c.164T>C | p.Leu55Pro | NA | Cytoplasmic | DC | PD | D-6.485 | 24.5 |  | (51) |
| 24 | c.221G>T | p.Ser74Ile | 56 | TM2 | DC | PD | D-5.536 | 26.6 | (52, 53) | (34), (35), (36), (37), (3), (7), (28) |
| 25 | c.251T>A | p.Ile84Asn | 2 | TM2 | DC | PD | D-5.554 | 24.9 |  | This study |
| 26 | c.307G>A | p.Asp103Asn | 7 | Extracellular | DC | PD | D-4.741 | 26.1 | (52) | (35), (5), (8), (16), (24) |
| 27 | c.319G>A | p.Asp107Asn | 3 | TM3 | DC | PD | D-4.835 | 25.6 | (52) | (30), (2) |
| 28 | c.320A>G | p.Asp107Gly | 1 | TM3 | DC | PD | D-6.77 | 25.8 | (52) | (51) |
| 29 | c.347G>T | p.Gly116Val | 4 | TM | DC | PD | D-3.283 | 24.4 | (52) | (5), (14) |
| 30 | c.360C>G | p.Ser120Arg | 3 | TM3 | DC | PD | D-3.467 | 22.6 | (52) | (15), (32) |
| 31 | c.376G>T | p.Ala126Ser | 1 | TM3 | DC | B | D-1.916 | 16.84 |  | (27) |
| 32 | c.382C>T | p.Arg128Cys | 3 | Cytoplasmic | DC | PD | D-7.805 | 29.4 | (52) | (34), (37), (22) |
| 33 | c.386A>G | p.Tyr129Cys | 1 | Cytoplasmic | DC | PD | D-8.884 | 28 | (52) | (23) |
| 34 | c.389T>A | p.Ile130Asn | NA | Cytoplasmic | DC | PD | D-6.356 | 28.6 |  | (54) |
| 35 | c.409C>T | p.Arg137Trp | 3 | Cytoplasmic | DC | PD | D-6.816 | 26 | (52, 53) | (5), (8) |
| 36 | c.410G>C | p.Arg137Pro | NA | Cytoplasmic | DC | PD | D-5.864 | 23.9 |  | (54) |
| 37 | c.415C>T | p.His139Tyr | NA | Cytoplasmic | DC | PD | D-5.799 | 24.4 |  | (54) |
| 38 | c.424G>T | p.Val142Leu | 6 | Cytoplasmic | P | B | N0.295 | 16.73 | (55) | (1), (5), (15) |
| 39 | c.427G>T | p.Thr143Ser | 2 | Cytoplasmic | DC | B | N0.295 | 25.6 | (55) | (5), (15) |
| 40 | c.433C>T | p.Arg145Cys | 2 | Cytoplasmic | DC | PD | D-6.194 | 27.7 |  | (10), (19) |
| 41 | c.437G>C | p.Arg146His | 12 | Cytoplasmic | DC | PD | D-4.559 | 27.6 | (52) | (34), (22), (26), (33) |
| 42 | c.455C>A | p.Thr152Lys | 2 | TM4 | P | B | N-1.219 | 9.268 | (52) | (21) |
| 43 | c.476C>G | p.Thr159Lys | 9 | TM4 | P | PD | D-2.906 | 7.277 | (52) | (35), (36), (25) |
| 44 | c.509A>T | p.His170Leu | NA | Extracellular | DC | PD | D-3.742 | 24.4 |  | (51) |
| 45 | c.539C>A | p.Ser180Ter | 2 | Extracellular | DC | NA | NA | 36 |  | (49) |
| 46 | c.573C>A | c.Cys191Ter | NA | TM5 | DC | NA | NA | 37 |  | (56) |
| 47 | c.593T>C | p.Leu198Pro | NA | TM5 | DC | PD | D-5.408 | 29.4 |  | (54) |
| 48 | c.601C>T | p.Arg201Ter | 1 | Cytoplasmic | DC | NA | NA | 41 |  | (32) |
| 49 | c.674T>G | p.Leu225Arg | 4 | TM6 | DC | PD | D5.227 | 27.8 | (52) | (5), (9) |
| 50 | c.676G>A | p.Gly226Arg | 3 | TM6 | DC | PD | D-7.553 | 31 | (52) | (24), (5) |
| 51 | c.697G>C | p.Ala233Pro | 7 | TM6 | DC | PD | D-3.929 | 26.5 |  | This study,(43), (15), (25) |
| 52 | c.712C>T | p.His238Tyr | 1 | TM6 | DC | PD | D-5.773 | 27.3 |  | (10) |
| 53 | c.742A>G | p.Ser247Gly | 1 | Extracellular | DC | B | N -1.947 | 22.7 | (57) | (31) |
| 54 | c.752G>T | p.Cys251Phe | 2 | Extracellular | DC | PD | D-10.587 | 27.5 | (52) | (30) |
| 55 | c.761A>G | p.Tyr254cys | 4 | Extracellular | DC | PD | D-7.581 | 27.4 | (52, 53) | (4), (29) |
| 56 | c.767C>T | p.Ser256Phe | NA | Extracellular | DC | PD | D-5.255 | 29.9 |  | (51) |
| 57 | c.818C>A | p.Pro273His | 1 | TM7 | DC | PD | D-8.321 | 28.7 | (52) | (28) |
| 58 | c.833T>G | p.Phen278Cys | 1 | TM7 | P | PD | D-6.107 | 32 |  | (23) |
| 59 | c.752G>A | p.Cys251Trp | 2 | Extracellular | DC | PD | D-10.587 | 27.0 |  | (43) |
|  | MRAP |  |  |  |  |  |  |  |  |  |
|  | Deletion |  |  |  |  |  |  |  |  |  |
| 1 | c.17_23delACGCCTC | p.Gln6Metfs*24 | 2 | TM | DC |  |  | 25.0 |  | (39) |
| 2 | c.88_90delAAG | p.Lys29del | 4 |  | DC |  |  | 21.6 |  | (43) |
| 3 | c.128delG | p.Val44Ter | 4 | TM | DC |  |  | 29.0 |  | (38), (40) |
|  | splice |  |  |  |  |  |  |  |  |  |
| 4 | c.106+3insT |  | 8 |  | P |  |  | 10.75 |  | (40), (43) |
| 5 | c.106+2_3dupTA |  | 2 |  | DC |  |  | 24.2 |  | (45) |
| 6 | c.106+1G>C |  | 6 |  | DC |  |  | 31 |  | (40) |
| 7 | c.106+1G>A |  | 2 |  | DC |  |  | 32 |  | (40) |
| 8 | c.106+1delG |  | 24 |  | DC |  |  | 23.9 |  | (40),(41),(42),(43) |
| 9 | c.106+1G>T |  | 2 |  | DC |  |  | 31 |  | (40) |
|  | Missense/nonsense |  |  |  |  |  |  |  |  |  |
| 10 | c.3G>A | p.Met1Ile | 18 | TM | DC | PD | N -2.188 | 24.0 |  | (46), (40) |
| 11 | c.33C>A | p.Tyr11Ter | NA | TM | DC | NA | NA | 35 |  |  |
| 12 | c.77T>C | p.Val26Ala | 2 | TM | DC | PD | D -3.834 | 25.7 |  | (44) |
| 13 | c.158T>C | p.Leu53Pro | 2 | TM | DC | PD | D -5.690 | 26.0 |  | (43) |
| 14 | c.175T>G | p.Tyr59Asp | 2 |  | P | PD | D -6.231 | 23.9 |  | (44) |

TM: Transmembrane; DC: Disease causing, PD: probably damaging, N: Neutral. P: Polymorphism, D: Deleterious, B: Benign

1. Chan LF, Campbell DC, Novoselova TV, Clark AJ, Metherell LA. Whole-Exome Sequencing in the Differential Diagnosis of Primary Adrenal Insufficiency in Children. Frontiers in endocrinology. 2015;6:113.

2. Delmas O, Marrec C, Caietta E, Simonin G, Morel Y, Girard N, et al. [Uncommon neonatal case of hypoglycemia: ACTH resistance syndrome]. Archives de pediatrie : organe officiel de la Societe francaise de pediatrie. 2014;21(12):1353-8.

3. Lin L, Hindmarsh PC, Metherell LA, Alzyoud M, Al-Ali M, Brain CE, et al. Severe loss-of-function mutations in the adrenocorticotropin receptor (ACTHR, MC2R) can be found in patients diagnosed with salt-losing adrenal hypoplasia. Clinical endocrinology. 2007;66(2):205-10.

4. Francescato G, Salvatoni A, Persani L, Agosti M. A rare genetic disorder causing persistent severe neonatal hypoglycaemia the diagnostic workup. BMJ case reports. 2012;2012.

5. Guran T, Buonocore F, Saka N, Ozbek MN, Aycan Z, Bereket A, et al. Rare Causes of Primary Adrenal Insufficiency: Genetic and Clinical Characterization of a Large Nationwide Cohort. The Journal of clinical endocrinology and metabolism. 2016;101(1):284-92.

6. Gujral J, Yau M, Yang AC, Kastury R, Romero CJ, Wallach E, et al. Primary Cortisol Deficiency and Growth Hormone Deficiency in a Neonate With Hypoglycemia: Coincidence or Consequence? Journal of the Endocrine Society. 2019;3(4):838-46.

7. Tsai SL, Green J, Metherell LA, Curtis F, Fernandez B, Healey A, et al. Primary Adrenocortical Insufficiency Case Series: Genetic Etiologies More Common than Expected. Hormone research in paediatrics. 2016;85(1):35-42.

8. Ishii T, Ogata T, Sasaki G, Sato S, Kinoshita EI, Matsuo N. Novel mutations of the ACTH receptor gene in a female adult patient with adrenal unresponsiveness to ACTH. Clinical endocrinology. 2000;53(3):389-92.

9. Akin MA, Akin L, Coban D, Ozturk MA, Bircan R, Kurtoglu S. A novel mutation in the MC2R gene causing familial glucocorticoid deficiency type 1. Neonatology. 2011;100(3):277-81.

10. Abuduxikuer K, Li ZD, Xie XB, Li YC, Zhao J, Wang JS. Novel Melanocortin 2 Receptor Variant in a Chinese Infant With Familial Glucocorticoid Deficiency Type 1, Case Report and Review of Literature. Frontiers in endocrinology. 2019;10:359.

11. Jacoby E, Barzilai A, Laufer J, Pade S, Anikster Y, Pinhas-Hamiel O, et al. Neonatal hyperpigmentation: diagnosis of familial glucocorticoid deficiency with a novel mutation in the melanocortin-2 receptor gene. Pediatric dermatology. 2014;31(1):e13-7.

12. Hirsch A, Meimaridou E, Fernandez-Cancio M, Pandey AV, Clemente M, Audi L, et al. Loss of the C terminus of melanocortin receptor 2 (MC2R) results in impaired cell surface expression and ACTH insensitivity. The Journal of clinical endocrinology and metabolism. 2011;96(1):E65-72.

13. Chan LF, Metherell LA, Krude H, Ball C, O'Riordan SM, Costigan C, et al. Homozygous nonsense and frameshift mutations of the ACTH receptor in children with familial glucocorticoid deficiency (FGD) are not associated with long-term mineralocorticoid deficiency. Clinical endocrinology. 2009;71(2):171-5.

14. Collares CV, Antunes-Rodrigues J, Moreira AC, Franca SN, Pereira LA, Soares MM, et al. Heterogeneity in the molecular basis of ACTH resistance syndrome. European journal of endocrinology. 2008;159(1):61-8.

15. Penhoat A, Naville D, El Mourabit H, Buronfosse A, Berberoglu M, Ocal G, et al. Functional relationships between three novel homozygous mutations in the ACTH receptor gene and familial glucocorticoid deficiency. Journal of molecular medicine (Berlin, Germany). 2002;80(7):406-11.

16. Kim CJ, Woo YJ, Kim GH, Yoo HW. Familial glucocorticoid deficiency with a point mutation in the ACTH receptor: a case report. Journal of Korean medical science. 2009;24(5):979-81.

17. Mazur A, Koehler K, Schuelke M, Skunde M, Ostanski M, Huebner A. Familial glucocorticoid deficiency type 1 due to a novel compound heterozygous MC2R mutation. Hormone research. 2008;69(6):363-8.

18. al Kandari HM, Katsumata N, al Alwan I, al Balwi M, Rasoul MS. Familial glucocorticoid deficiency in five Arab kindreds with homozygous point mutations of the ACTH receptor (MC2R): genotype and phenotype correlations. Hormone research in paediatrics. 2011;76(3):165-71.

19. Aza-Carmona M, Barreda-Bonis AC, Guerrero-Fernandez J, Gonzalez-Casado I, Gracia R, Heath KE. Familial glucocorticoid deficiency due to compound heterozygosity of two novel MC2R mutations. Journal of pediatric endocrinology & metabolism : JPEM. 2011;24(5-6):395-7.

20. Tsiotra PC, Koukourava A, Kaltezioti V, Geffner ME, Naville D, Begeot M, et al. Compound heterozygosity of a frameshift mutation in the coding region and a single base substitution in the promoter of the ACTH receptor gene in a family with isolated glucocorticoid deficiency. Journal of pediatric endocrinology & metabolism : JPEM. 2006;19(9):1157-66.

21. Turan S, Hughes C, Atay Z, Guran T, Haliloglu B, Clark AJ, et al. An atypical case of familial glucocorticoid deficiency without pigmentation caused by coexistent homozygous mutations in MC2R (T152K) and MC1R (R160W). The Journal of clinical endocrinology and metabolism. 2012;97(5):E771-4.

22. Weber A, Toppari J, Harvey RD, Klann RC, Shaw NJ, Ricker AT, et al. Adrenocorticotropin receptor gene mutations in familial glucocorticoid deficiency: relationships with clinical features in four families. The Journal of clinical endocrinology and metabolism. 1995;80(1):65-71.

23. Chan LF, Chung T-T, Massoud AF, Metherell LA, Clark AJ. Functional consequence of a novel Y129C mutation in a patient with two contradictory melanocortin-2-receptor mutations. European journal of endocrinology. 2009;160(4):705.

24. Amano N, Narumi S, Hayashi M, Takagi M, Imai K, Nakamura T, et al. Genetic defects in pediatric-onset adrenal insufficiency in Japan. European journal of endocrinology. 2017;177(2):187-94.

25. Bizzarri C, Olivini N, Pedicelli S, Marini R, Giannone G, Cambiaso P, et al. Congenital primary adrenal insufficiency and selective aldosterone defects presenting as salt-wasting in infancy: a single center 10-year experience. Italian journal of pediatrics. 2016;42(1):73.

26. Matsuura H, Shiohara M, Yamano M, Kurata K, Arai F, Koike K. Novel compound heterozygous mutation of the MC2R gene in a patient with familial glucocorticoid deficiency. Journal of Pediatric Endocrinology and Metabolism. 2006;19(9):1167-70.

27. Artigas RA, Gonzalez A, Riquelme E, Carvajal CA, Cattani A, Martínez-Aguayo A, et al. A novel adrenocorticotropin receptor mutation alters its structure and function, causing familial glucocorticoid deficiency. The Journal of Clinical Endocrinology & Metabolism. 2008;93(8):3097-105.

28. Wu SM, Stratakis CA, Chan CH, Hallermeier KM, Bourdony CJ, Rennert OM, et al. Genetic heterogeneity of adrenocorticotropin (ACTH) resistance syndromes: identification of a novel mutation of the ACTH receptor gene in hereditary glucocorticoid deficiency. Molecular genetics and metabolism. 1998;64(4):256-65.

29. Tsigos C, Arai K, Latronico AC, DiGeorge AM, Rapaport R, Chrousos GP. A novel mutation of the adrenocorticotropin receptor (ACTH-R) gene in a family with the syndrome of isolated glucocorticoid deficiency, but no ACTH-R abnormalities in two families with the triple A syndrome. The Journal of clinical endocrinology and metabolism. 1995;80(7):2186-9.

30. Naville D, Barjhoux L, Jaillard C, Faury D, Despert F, Esteva B, et al. Demonstration by transfection studies that mutations in the adrenocorticotropin receptor gene are one cause of the hereditary syndrome of glucocorticoid deficiency. The Journal of clinical endocrinology and metabolism. 1996;81(4):1442-8.

31. Hiroi N, Yakushiji F, Shimojo M, Watanabe S, Sugano S, Yamaguchi N, et al. Human ACTH hypersensitivity syndrome associated with abnormalities of the ACTH receptor gene. Clinical endocrinology. 1998;48(2):129-34.

32. Tsigos C, Arai K, Hung W, Chrousos GP. Hereditary isolated glucocorticoid deficiency is associated with abnormalities of the adrenocorticotropin receptor gene. The Journal of clinical investigation. 1993;92(5):2458-61.

33. Slavotinek AM, Hurst JA, Dunger D, Wilkie AO. ACTH receptor mutation in a girl with familial glucocorticoid deficiency. Clinical genetics. 1998;53(1):57-62.

34. Weber A, Clark A, Perry L, Honour J, Savage M. Diminished adrenal androgen secretion in familial glucocorticoid deficiency implicates a significant role for ACTH in the induction of adrenarche. Clinical endocrinology. 1997;46(4):431-7.

35. Elias LL, Huebner A, Metherell LA, Canas A, Warne GL, Bitti MLM, et al. Tall stature in familial glucocorticoid deficiency. Clinical endocrinology. 2000;53(4):423-30.

36. Selva KA, LaFranchi SH, Boston B. A novel presentation of familial glucocorticoid deficiency (FGD) and current literature review. J Pediatr Endocrinol Metab. 2004;17(1):85-92.

37. Weber A, JL Clark A. Mutations of the ACTH receptor gene are only one cause of familial glucocorticoid deficiency. Human Molecular Genetics. 1994;3(4):585-8.

38. Rumie H, Metherell L, Clark A, Beauloye V, Maes M. Clinical and biological phenotype of a patient with familial glucocorticoid deficiency type 2 caused by a mutation of melanocortin 2 receptor accessory protein. European journal of endocrinology. 2007;157(4):539-42.

39. Modan-Moses D, Ben-Zeev B, Hoffmann C, Falik-Zaccai TC, Bental YA, Pinhas-Hamiel O, et al. Unusual presentation of familial glucocorticoid deficiency with a novel MRAP mutation. The Journal of Clinical Endocrinology & Metabolism. 2006;91(10):3713-7.

40. Metherell LA, Chapple JP, Cooray S, David A, Becker C, Rüschendorf F, et al. Mutations in MRAP, encoding a new interacting partner of the ACTH receptor, cause familial glucocorticoid deficiency type 2. Nature genetics. 2005;37(2):166-70.

41. Akın L, Kurtoğlu S, Kendirici M, Akın MA. Familial glucocorticoid deficiency type 2: a case report. Journal of clinical research in pediatric endocrinology. 2010;2(3):122.

42. Chen C, Zhou R, Fang Y, Jiang L, Liang L, Wang C. Neonatal presentation of familial glucocorticoid deficiency with a MRAP mutation: a case report. Molecular genetics and metabolism reports. 2016;9:15-7.

43. Guran T, Buonocore F, Saka N, Ozbek MN, Aycan Z, Bereket A, et al. Rare causes of primary adrenal insufficiency: genetic and clinical characterization of a large nationwide cohort. The Journal of Clinical Endocrinology & Metabolism. 2016;101(1):284-92.

44. Hughes C, Chung T, Habeb A, Kelestimur F, Clark A, Metherell L. Missense mutations in the melanocortin 2 receptor accessory protein that lead to late onset familial glucocorticoid deficiency type 2. The Journal of Clinical Endocrinology & Metabolism. 2010;95(7):3497-501.

45. Jain V, Metherell L, David A, Sharma R, Sharma P, Clark A, et al. Neonatal presentation of familial glucocorticoid deficiency resulting from a novel splice mutation in the melanocortin 2 receptor accessory protein. European journal of endocrinology. 2011;165(6):987.

46. Collares CVA, Antunes-Rodrigues J, Moreira AC, Franca SN, Pereira LA, Soares MMS, et al. Heterogeneity in the molecular basis of ACTH resistance syndrome. European journal of endocrinology. 2008;159(1):61-8.

47. Chan L, Metherell L, Naville D. A novel mutation of MRAP (melanocortin 2 receptor accessory protein) gene in two brothers with familial glucocorticoid deficiency. Endocrine Abstracts The Endocrine Society, Boston, poster. 2006(6).

48. Mazur A, Koehler K, Schuelke M, Skunde M, Ostański M, Huebner A. Familial glucocorticoid deficiency type 1 due to a novel compound heterozygous MC2R mutation. Hormone Research in Paediatrics. 2008;69(6):363-8.

49. Chan LF, Metherell LA, Krude H, Ball C, O'Riordan SM, Costigan C, et al. Homozygous nonsense and frameshift mutations of the ACTH receptor in children with familial glucocorticoid deficiency (FGD) are not associated with long‐term mineralocorticoid deficiency. Clinical endocrinology. 2009;71(2):171-5.

50. Metherell LA, Chan LF, Clark AJ. The genetics of ACTH resistance syndromes. Best Pract Res Clin Endocrinol Metab. 2006;20(4):547-60.

51. Clark AJ, Metherell LA, Cheetham ME, Huebner A. Inherited ACTH insensitivity illuminates the mechanisms of ACTH action. Trends Endocrinol Metab. 2005;16(10):451-7.

52. Chung T, Webb T, Chan L, Cooray S, Metherell L, King P, et al. The majority of adrenocorticotropin receptor (melanocortin 2 receptor) mutations found in familial glucocorticoid deficiency type 1 lead to defective trafficking of the receptor to the cell surface. The Journal of Clinical Endocrinology & Metabolism. 2008;93(12):4948-54.

53. Fluck CE, Martens JW, Conte FA, Miller WL. Clinical, genetic, and functional characterization of adrenocorticotropin receptor mutations using a novel receptor assay. The Journal of clinical endocrinology and metabolism. 2002;87(9):4318-23.

54. Naville D, Penhoat A, Begeot M. [ACTH resistance syndromes]. Ann Endocrinol (Paris). 2000;61(5):428-39.

55. Chen M, Aprahamian CJ, Kesterson RA, Harmon CM, Yang Y. Molecular identification of the human melanocortin-2 receptor responsible for ligand binding and signaling. Biochemistry. 2007;46(40):11389-97.

56. Mueller OT, Coovadia A. Novel human pathological mutations. Gene symbol: MC2R. Disease: glucocorticoid deficiency. Human genetics. 2010;127(4):479.

57. Swords FM, Noon LA, King PJ, Clark AJ. Constitutive activation of the human ACTH receptor resulting from a synergistic interaction between two naturally occurring missense mutations in the MC2R gene. Molecular and cellular endocrinology. 2004;213(2):149-54.
